# Triphenylantimony(V) Catecholates of the Type (3-RS-4,6-DBCat)SbPh_3_-Catechol Thioether Derivatives: Structure, Electrochemical Properties, and Antiradical Activity

**DOI:** 10.3390/molecules26082171

**Published:** 2021-04-09

**Authors:** Ivan V. Smolyaninov, Georgy K. Fukin, Nadezhda T. Berberova, Andrey I. Poddel’sky

**Affiliations:** 1 Department of Chemistry, Astrakhan State Technical University, 16 Tatisheva Str., 414056 Astrakhan, Russia; ivsmolyaninov@gmail.com (I.V.S.); nberberova@gmail.com (N.T.B.); 2 G.A. Razuvaev Institute of Organometallic Chemistry, Russian Academy of Sciences, 49 Tropinina Str., 603137 Nizhny Novgorod, Russia; gera@iomc.ras.ru

**Keywords:** redox-active ligand, catechol, antimony, X-ray, cyclic voltammetry, antiradical activity

## Abstract

A new series of triphenylantimony(V) 3-alkylthio/arylthio-substituted 4,6-di-tert-butylcatecholates of the type (3-RS-4,6-DBCat)SbPh_3_, where R = n-butyl (**1**), n-hexyl (**2**), n-octyl (**3**), cyclopentyl (**4**), cyclohexyl (**5**), benzyl (**6**), phenyl (**7**), and naphthyl-2 (**8**), were synthesized from the corresponding catechol thioethers and Ph_3_SbBr_2_ in the presence of a base. The crystal structures of **1**, **2**, **3**, and **5** were determined by single-crystal X-ray analysis. The coordination polyhedron of **1**–**3** is better described as a tetragonal pyramid with a different degree of distortion, while that for **5**- was a distorted trigonal bipyramid (*τ* = 0.014, 0.177, 0.26, 0.56, respectively). Complexes demonstrated different crystal packing of molecules. The electrochemical oxidation of the complexes involved the catecholate group as well as the thioether linker. The introduction of a thioether fragment into the aromatic ring of catechol ligand led to a shift in the potential of the “catechol/o-semiquinone” redox transition to the anodic region, which indicated the electron-withdrawing nature of the RS group. The radical scavenging activity of the complexes was determined in the reaction with DPPH radical.

## 1. Introduction

Redox-active quinoid compounds have been widely investigated as anti-cancer, anti-bacterial, anti-inflammatory, and anti-parasitic agents [1,2,3,4,5,6]. Such compounds demonstrate a wide spectrum of pharmacological activity [7,8,9] and behave both as antioxidants [10,11,12,13,14,15] and prooxidants [16,17,18,19]. Functionalization of o-quinones/catechols allows the expansion of their redox potential and biochemical activity as well as the modification of coordinating ability, etc. [20,21,22,23,24,25]. Catechol-thioethers are prospective objects from the viewpoint of studying the antioxidant properties of polyfunctional compounds, and are new objects in coordination chemistry [26,27,28,29,30,31,32]. For example, complexes of 4,6-di-tert-butylcatechol derivatives containing cystamine or cysteine residues at position 3 of such ligands are actively studied [26,27]. Bifunctional compounds combining a catechol moiety and a free thiol group have both chelating properties and the ability to adsorb on a surface. The thio linker also allows the formation of covalent bonds with a metal, in particular with gold [33]. Cobalt complexes with a thiol-functionalized catecholate ligand are able to undergo valence tautomerism [34].

Metal compounds based on sulfur-containing catecholate/o-semiquinolate ligands have biological activity. Hence, copper(II) complexes of the type CuL_2_ with 4,6-di-tert-butyl-3-(2-hydroxyethylsulfanyl)-1,2-benzenedithiol and 2-[4,6-di-tert-butyl-2,3-dihydroxyphenylsulfanyl] acetic acid exhibit fungicidal properties [35]. Iron(II), copper(II), and related cobalt(II), nickel(II), and zinc(II) complexes based on 2-(4,6-di-tert-butyl-2,3-dihydroxyphenylsulfanyl)acetic and 2-(4,6-di-tert-butyl-2,3-dihydroxyphenylsulphenyl)acetic acids possess remarkable antibacterial activity [36]. Complexes of copper(II), cobalt(II), and nickel(II) with 4,6-di-tert-butyl-3-(2-hydroxyethylsulfanyl)-1,2-benzenedithiol, along with their fungicidal activity, inhibit the replication of the human immunodeficiency virus [37].

The coordination chemistry of o-quinoid type ligands is an extensively developed area [38,39,40,41,42,43]. Redox-active ligands allow for significant expansion of the reactivity of transition and main group metal complexes bearing this type of ligand.

Antimony(III/V) coordination and organometallic compounds have attracted the attention of researchers due to the possibility of their application in the design of functional organic materials capable of binding anions and “small” molecules (O_2_, CO_2_), behaving as chemosensors, catalysts, and potential therapeutic agents [44,45].

Recently it was discovered that metal-coordinated stibines can exhibit “non-innocent” behavior: two-electron redox transformations of an antimony atom affect the interaction “antimony-metal” in reaction with halogens, which makes it possible to use such compounds as chemosensors [46,47,48,49]. Prof. F. Gabbaï has shown that the catecholate-containing Sb(V)-Pt complex is also active toward the fluoride anion [50]. The scientific group led by F. Gabbaï has investigated a whole series of antimony catecholate compounds that have a high selectivity to the binding of fluoride anion at concentrations less than 1 ppm [51,52,53]. Antimony(V) organometallic derivatives with catecholate ligands are the basis for the design of selective sensors for the fluoride anion due to the ability of neutral stiboranes to play a role as Lewis acids. Indeed, in a number of works it has been shown that organometallic antimony(V) catecholates are Lewis acids and can form adducts with various nucleophilic agents [54,55,56,57]. Heterometallic complexes with antimony(V) catecholate as a ligand reveal a catalytic activity in some perspective organic reactions. Heteronuclear catecholato-bis-(phosphino)antimony-gold complex possesses catalytic activity in hydroamination reactions of phenylacetylene with anilines or cyclization of N-(prop-2-ine-1-yl)benzamide [58]. Catecholate complexes of antimony(V), being Lewis acids, are active not only in the binding of bases, but also catalyze hydrogen transfer reactions in the reduction of N-benzylideneaniline and quinoline, and also participate in the reaction of diphenylbromomethane with water and acetonitrile to form N-benzhydrylacetamide [59]. The introduction of electron-withdrawing fluorine atoms into phenyl groups at the antimony atom increases significantly the Lewis acidity of antimony(V) catecholate complexes, which allows for the binding of triethylphosphine oxide. Antimony(V) catecholate can bind formaldehyde into the corresponding complex, which opens prospects for molecular recognition and colorimetric detection of formaldehyde in aqueous solutions [60].

Earlier, we found that antimony(V) complexes can mimic the chemical behavior of transition metal complexes with activity toward O_2_. The interaction of triphenylantimony(V) o-amidophenolates and some catecholates with O_2_ leads to the formation of spiroendoperoxide complexes [61,62,63,64]. The reaction mechanism involves a change in the oxidation state of the o-amidophenodianionic redox-active ligand to radical anion and coordination of the superoxide radical anion to antimony(V) atom, followed by recombination of the radical centers. In contrast to the transition metals, the O_2_ binding does not lead to a change in the antimony oxidation state, while the redox active ligand is involved in the redox reaction. The nature of functional groups in redox active ligands [65,66,67,68], as well as in substituents at a central antimony atom [69,70,71,72,73,74], affects the redox properties of antimony catecholates and their antioxidant activity [75,76,77,78,79,80]. Electron-donating groups in catecholate or at the antimony atom in complexes of the type CatSbR_3_ shift the oxidation potential to the anodic region. Conversely, the presence of electron-withdrawing fragments in the catecholate ligand should hinder the oxidation of such compounds, prevent the formation of spiroendoperoxides, and reduce the antioxidant activity.

The presence of a catecholate fragment bound to triphenylantimony(V) in the polymer structure allows the creation of oxygen-sensitive materials [81,82,83].

In the present paper we report the structure and electrochemical properties of triphenylantimony(V) 3-alkylthio/arylthio-substituted 4,6-di-tert-butylcatecholates and radical scavenging activity in the DPPH (2,2-diphenyl-1-picrylhydrazyl) test.

## 2. Results and Discussion

### 2.1. Synthesis and Characterization

**Scheme 1:**
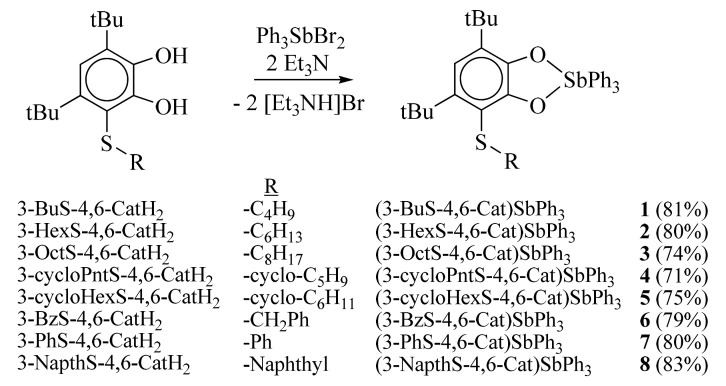
Synthesis of complexes **1**–**8**.

The antimony(V) catecholates **1**–**8** were synthesized with preparative yields of 71–83% by the exchange reaction of the corresponding 3-alkylthio/arylthio-4,6-di-tert-butylcatechols with triphenylantimony(V) dibromide in the presence of triethylamine as a base (Scheme 1). This method is more laborious and not as simple as the direct oxidative addition of o-benzoquinones to stibines, but the reason for its application in this case is that the preparation of the corresponding 3-organylthio-substituted o-benzoquinones proceeds with low yields of the corresponding o-benzoquinones, and moreover, they are photosensitive.

All new catecholates **1**–**8** were characterized by means of IR-, ^1^H and ^13^C-NMR spectroscopy and by elemental analysis.

### 2.2. X-ray Structures

**Figure 1:**
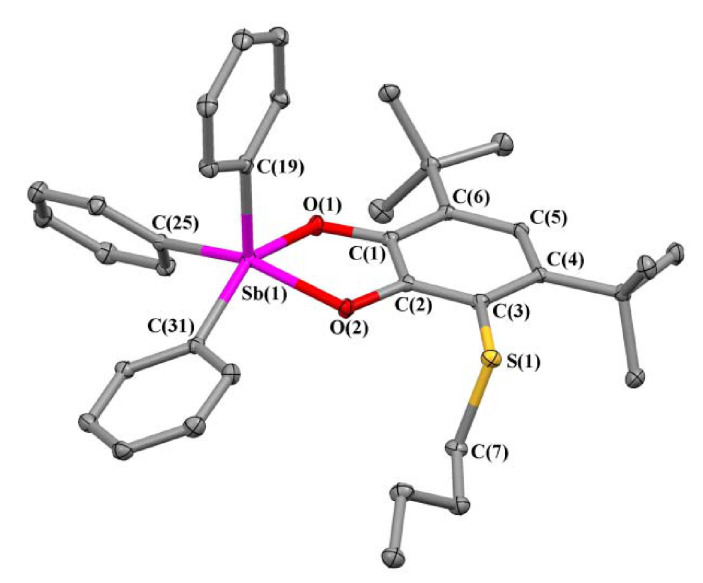
The X-ray structure of (3-BuS-4,6-Cat)SbPh_3_ (**1**). Hydrogen atoms are omitted, ellipsoids of 30% probability.

**Figure 2:**
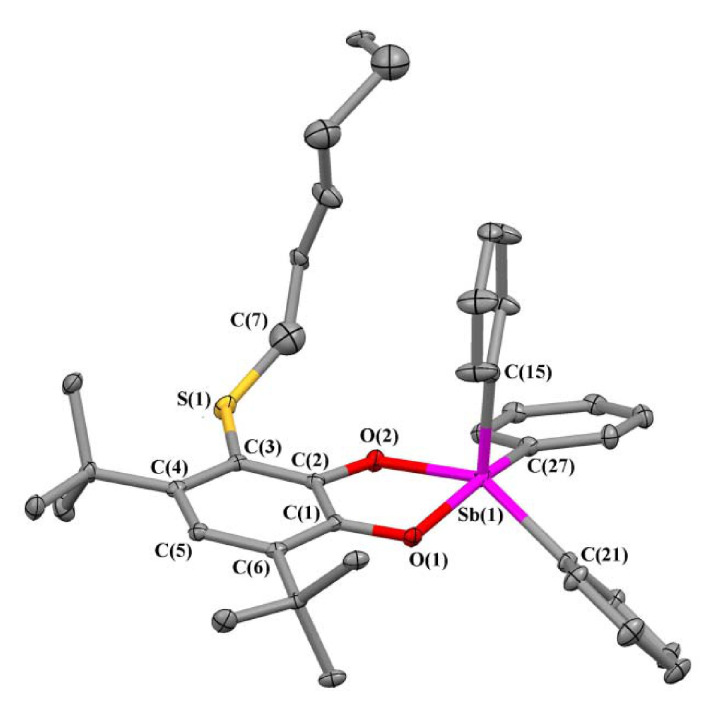
The X-ray structure of (3-HexS-4,6-Cat)SbPh_3_ (**2**). Hydrogen atoms are omitted, ellipsoids of 30% probability.

**Figure 3:**
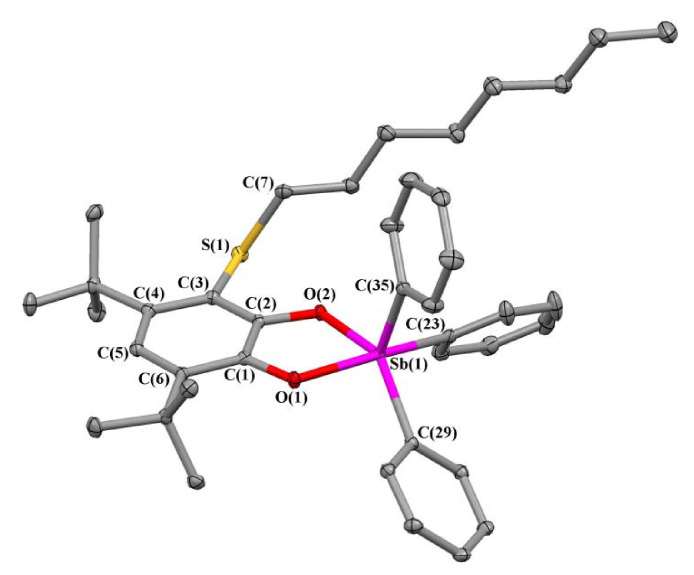
The X-ray structure of (3-OctS-4,6-Cat)SbPh_3_ (**3**). Hydrogen atoms are omitted, ellipsoids of 30% probability.

**Figure 4:**
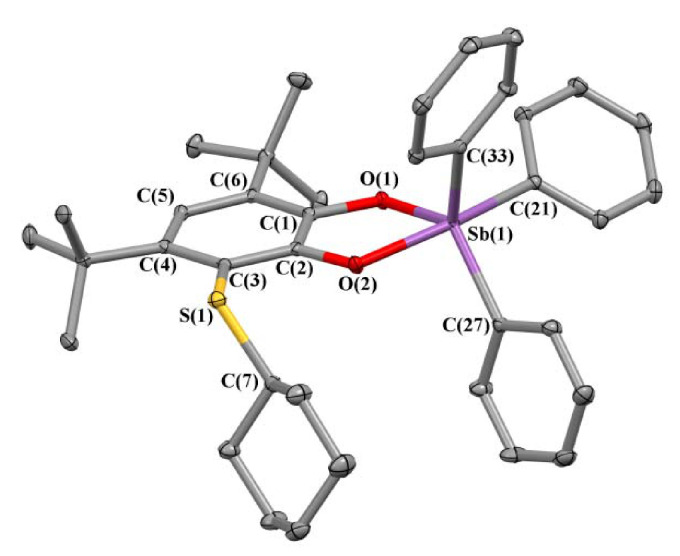
The X-ray structure of (3-cycloHexS-4,6-Cat)SbPh_3_ (**5**). Hydrogen atoms are omitted, ellipsoids of 30% probability.

The molecular structures of **1**, **2**, **3**, and **5** in crystalline state were determined by single-crystal X-ray analysis (Figure 1, Figure 2, Figure 3 and Figure 4). The selected bond distances and angles are listed in Table 1. The X-ray experimental details are given in Table S1.

The central antimony atom in complexes **1**–**3** is arranged by a distorted tetragonal pyramidal environment that is confirmed by differences in the basal and apical Sb-C bond distances: the apical bonds Sb(1)-C(19) (**1**), Sb(1)-C(15) (**2**), and Sb(1)-C(35) (**3**) are 0.025–0.040 Å shorter than the corresponding basal bonds Sb-C (Table 1). The distortion of the coordination polyhedron from the tetragonal pyramid to the trigonal bipyramid increases in a row **1**-**2**-**3**-**5**: the values of parameter *τ* describing the polyhedron of pentacoordinated compounds are the following: 0.014 (**1**), 0.177 (**2**), 0.26 (**3**), and 0.56 (**5**). The parameter *τ* = 0 in an ideal tetragonal pyramid; *τ* = 1 in an ideal trigonal bipyramid [84]. The geometric characteristics of redox-active O,O’-chelating ligands (such as the distances of oxygen-carbon bonds O(1)-C(1) and O(2)-C(2) and carbon-carbon bonds in a six-membered carbon cycle С(1-6)) correspond to the single oxygen-carbon bonds in catecholato complexes of antimony [85,86,87,88,89,90,91,92,93,94,95,96] and other metals [97,98,99,100,101,102].

It would be interesting to mention different packing motifs in crystals of these complexes. Molecules (6-BuS-3,5-DBCat)SbPh_3_ (**1**) form linear chains by means of intermolecular interactions of T-type “CH_Ph_…π-system_Cat_” between carbon atom С(22) in para-position of an apical phenyl group and aromatic π-system of catecholate; the corresponding distance is 3.54 Å (Figure S1).

In crystal of (6-HexS-3,5-DBCat)SbPh_3_ (**2**), molecules form the related chains (Figure S2) with the distance 3.69(1) Å between carbon atoms C(24) in the para-position of the basal phenyl group (the phenyl group at the base of the pyramid) located in the trans-position to the oxygen atom O(2). Such chains are bound to each other by the additional intermolecular interactions between the central antimony atoms and carbons C(31) in the meta-position of the second basal phenyl groups (the Sb(1)-C(31) distances are 3.67 Å).

Molecules (6-OctS-3,5-DBCat)SbPh_3_ (**3**) in crystal form layers (Figure S3) where they are pair-wise bound by the T-type interactions “CH_Ph_…π-system_Cat_”. The corresponding distances between the C(38) atom in the para-position of the apical phenyl group and the plane of the aromatic ring of the catecholate ligand are 3.47 Å. Between two neighboring pairs in a chain, intermolecular contacts are observed between the central antimony atoms and carbon atoms C(31) in the meta-position of one of the basal phenyl groups (the intermolecular distances Sb(1)...C(31) are 4.18(1) Å). The layers are arranged in such a way that they face each other with long octyl groups, so the layers are separated by interlayers of octyl substituents.

The packing motif of (3-cycloHexS-4,6-DBCat)SbPh_3_ (**5**) differs from those for **1**–**3**. Complex molecules are packed in pairs also by the T-type interactions “CH_Ph_…π-system_Cat_” (Figure S4) (the distance between the C(31) atom in the meta-position of one basal phenyl group and the plane of the aromatic ring of the catecholate ligand is 3.54 Å). However, in this case there are no remarkable additional interactions between the neighboring pairs. The close situation was observed in crystals of triphenylantimony(V) 3-alkoxy-4,6-di-tert-butylcatecholates and 4,5-dialkoxy-3,6-di-tert-butylcatecholates [68,103,104].

### 2.3. Cyclic Voltammetry

**Figure 5:**
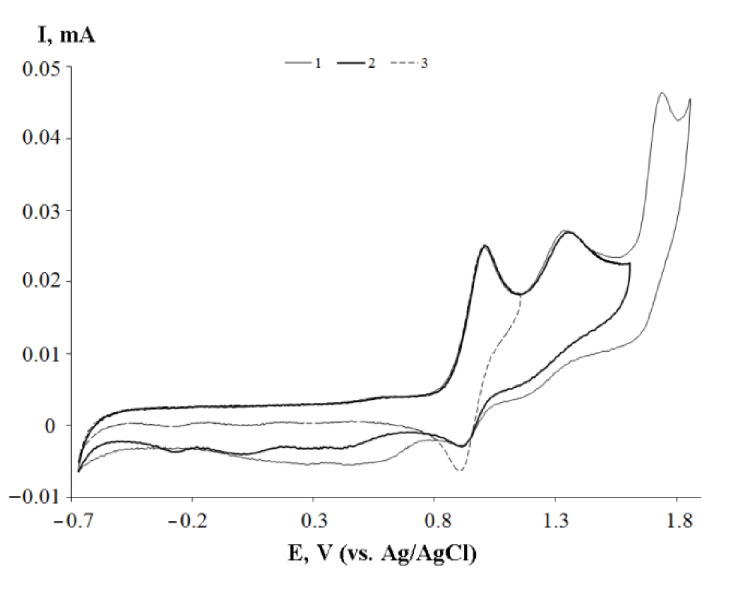
The CV curves of the oxidation of **7** in potential ranges from −0.68 to 1.85 V (curve 1); from −0.68 to 1.60 V (curve 2); from −0.68 to 1.15 В (curve 3); (CH_2_Cl_2_, GC anode, Ag/AgCl/KCl(sat.), 0.15M [nBu_4_N]ClO_4_, C = 2·10^−3^ M, argon).

**Scheme 2:**
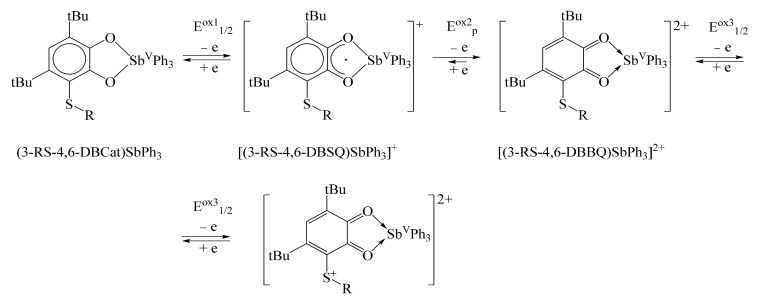
Synthesis of complexes **1**–**8**.

For the complexes obtained, the electrochemical properties were investigated by the cyclic voltammetry (CV) method (Table 2). In contrast to the triphenylantimony(V) catecholate complexes with alkoxy-, halide substituents in Cat ligand, compounds **1**–**8** are characterized by the presence of three oxidation stages (Figure 5, Figures S5–S12), corresponding to the two-stage oxidation of the catecholate fragment (Scheme 2) and oxidation of the thioether group.

As in the case of free catechol ligands [28,29], the presence of an electron-withdrawing sulfur atom at position 3 of Cat ligand in **1**–**8** leads to a shift of the first oxidation potential of the complexes, which characterizes the oxidation stage “catecholate/o-semiquinone”, to the anodic region by 0.06–0.08 V as compared with the related triphenylantimony(V) 3,6-di-tert-butylcatecholate [66,72]. In contrast to the pronounced donor effect of RO-substituents in the redox-active catecholate ligand [75,80], the introduction of the RS-group complicates the oxidation process of the complexes and indicates its electron-withdrawing effect. So, these catecholates **1**–**8** do not tend to bind molecular oxygen due to high anodic potential of the oxidation potential “catecholate/o-semiquinone” (e.g., in contrast to that for triphenylantimony(V) 4-methoxy- or 4,5-dimethoxy-3,6-di-tert-butylcatecholates [75,80]).

It is worthy to note a decrease of the current ratio, which indicates a decrease in the stability of electrogenerated monocationic species [(3-RS-4,6-DBSQ)SbPh_3_]^+^. A similar, but more pronounced, effect was observed for antimony(V) catecholates with chlorine atoms in the 4th and 5th positions of the catecholate ring [66,105].

As a result of a pulse potential sweep (to 1.2 V), a quasi-reversible peak of the reduction of o-benzoquinones (−0.42 to −0.51 V) formed as a result of partial destruction of electrogenerated monocationic complexes can be observed in the cathode region (Figure S10).

For most compounds, the values of E^ox1^_1/2_ and E^ox2^_p_ are fixed in a narrow potential range. However, the presence of phenyl or naphthyl substituents at the sulfur atom in the case of catecholates **7** and **8** favors the shift of the potential of the second stage (“o-semiquinone/o-benzoquinone”) to the cathodic region. In the case of compounds **7** and **8**, the second quasi-reversible oxidation peaks are observed (I_c_/I_a_ = 0.4–0.6), which indicates a partial stabilization of electrogenerated dicationic form [(3-RS-4,6-DBBQ)SbPh_3_]^2+^ (Figure 5).

The third quasi-reversible anodic peak is also observed at the close potentials and corresponds to the oxidation of the thioether linker. The observed values of Е^ox3^_1/2_ are practically identical to these potentials for free catechol ligands in dichloromethane (1.67–1.70 V). For compounds **7** and **8**, the third redox wave is irreversible. A significant decrease in the reversibility of the first anodic peak with the expansion of the potential sweep to 1.9 V suggests a partial destruction of the complex with the release of the o-benzoquinone ligand. It should be noted that the current intensities of the third anodic peaks of complexes, as well as for free ligands, depend on the substituent at the sulfur atom: a two-electron level was observed for compounds **3** and **8**, while a one-electron level was found for compounds **4**–**7**.

Thus, the introduction of a thioether linker into the structure of redox-active catecholate ligands makes it possible to expand the number of possible redox states of antimony(V) catecholato complexes due to the participation of RS-organic fragments in redox reactions.

### 2.4. Antiradical Activity Test with DPPH

We have previously shown that triarylantimony (V) complexes with catecholate ligands act as effective traps and can not only intercept molecular oxygen, but also neutralize alkylperoxy radicals and DPPH, promote the destruction of hydroperoxides, and inhibit lipid peroxidation processes [75,76,77,78,79,80]. In the continuation of our research, it was interesting to evaluate the effect of the RS group on the radical scavenging activity in the reaction with the stable DPPH radical. The data on the investigation of the antiradical activity of triphenylantimony(V) catecholates **1**–**8** are given in Table 3.

The values of EC_50_ and TEC_50_ indicate a decrease in the antiradical activity of complexes **1**–**8** in comparison with triphenylantimony(V) 3,6-di-tert-butylcatecholate and (4-MeO-3,6-Cat)SbPh_3_ [75]. A feature of compounds **1**–**8** is a higher anodic potential of the “catecholate/o-benzosemiquinone” redox transition, which causes a decrease in the activity of these complexes in the electron transfer reaction to the DPPH radical. The number of converted DPPH molecules for compounds **1**–**8** is less than one, in contrast to those for complexes (3,6-Cat)SbPh_3_ and (4-MeO-3,6-Cat)SbPh_3_ (the numbers are 1.50 and 3.7, respectively) [75]. The AE action index also suggests reduced efficiency of the antiradical action of the complexes under the given experimental conditions. In general, the introduction of an alkylthio group into the catecholate ring leads to a significant decrease of antiradical activity in the test with the DPPH radical. However, the presence of a thioether linker can modulate the antioxidant activity of the complexes when interacting with hydrogen peroxide and organic peroxides, because this group is a secondary antioxidant.

## 3. Materials and Methods

### 3.1. General Remarks

For the synthesis of the compounds, commercial reagents from Sigma-Aldrich, Acros Organics, Merck, Alfa Aesar were used without additional purification. The used solvents were purified and dried by known methods [106]. The ^1^H, ^13^C-NMR spectra were recorded using spectrometers Bruker AVANCE DPX-200 (for **1**–**7**) and Bruker Avance III (400 MHz) (for **8**) with solvent CDCl_3_; chemical shifts are given in ppm on the δ scale relative to the internal standard, tetramethylsilane. The IR spectra were recorded on an FSM 1201 FTIR spectrometer (LLC “Monitoring”, Saint Petersburg, Russia) in KBr pellets. Elemental analysis (C, H) was performed on Euro EA 3000 and Analytik Jena multi EA 5000 (C, S) elemental analyzers. The sulfur content was determined using an ASE-1 X-ray fluorescence analyzer.

### 3.2. X-ray Diffraction Studies

The X-ray diffraction data were collected on a Smart Apex (**1**–**3**) and an Agilent Xcalibur E (**5**) diffractometer (Mo-*K*_α_ radiation, *ω*- and *φ*-scan technique, *λ* = 0.71073 Å). The intensity data were integrated by SAINT [107] (for **1**–**3**) and CrysAlisPro [108] (for **5**). SADABS ([109] for **1**–**3**) and SCALE3 ABSPACK ([110] for **5)** were used to perform area-detector scaling and absorption corrections. All structures were solved by the dual-space method using the SHELXT program [111]. All structures were refined on *F*^2^_hkl_ using the SHELXTL package [112]. All non-hydrogen atoms were refined anisotropically. Hydrogen atoms were placed in calculated positions and refined in the riding model. Crystallographic data for the complexes, as well as the main parameters of the X-ray diffraction experiment and refinement of structures are given in Table S1. CCDC 2071115-2071118 for **1, 2**∙0.5Toluene, **3** and **5**, respectively, contain the supplementary crystallographic data for this paper. These data can be obtained free of charge from The Cambridge Crystallographic Data Centre via http://www.ccdc.cam.ac.uk/data_request/cif (accessed on 26 February 2021).

### 3.3. Cyclic Voltammetry

The oxidation potentials were measured by the CV method in a three-electrode cell using an IPC-pro potentiostat (STF “Volta”, Saint Petersburg, Russia) in an argon atmosphere. The working electrode was a stationary glassy carbon electrode with a diameter of 2 mm, and the auxiliary electrode was a platinum plate. The reference electrode used -Ag/AgCl/ KCl(sat.) with a waterproof diaphragm. Potential sweep rate was 0.2 V∙s^−1^. All measurements were carried out under argon. The samples were dissolved in the pre-deaerated solvent. The scan rate (ν) was 200 mV∙s^−1^. The supporting electrolyte 0.15 M Bu_4_NClO_4_ (99%, electrochemical grade, Fluka) was dried in vacuum (48 h) at 50 °C. The concentration of complexes was 1–3 mmol.

### 3.4. DPPH Test

DPPH radical scavenging activity was performed according to the method of Bondet et al. [113]. The interaction of the studied compounds with the DPPH radical was carried out in dichloromethane at 298 K in the concentration range from 5 to 50 µm. The initial concentration of DPPH radical was 50 μmol, with the extinction coefficient of the radical in CH_2_Cl_2_ calculated (λ_max_ = 527 nm; ε_max_ = 16700 M^−1^∙cm^−1^).

The determination of EC_50_ was performed by the dependence of the residual concentration of the stable radical on the molar ratio, expressed by the number of antioxidant moles per one mol of the stable radical. The parameter EC_50_ is the ratio of the concentration of the antioxidant required to reduce the amount of DPPH radical by 50% from the initial value. The parameter TEC_50_ is the time necessary to reach equilibrium at an antioxidant concentration equal to the EC_50_. The number of the converted molecules of radical was calculated by the formula n_DPPH_ = С_0_(DPPH)/2·EC_50_, where С_0_(DPPH) is an initial concentration of DPPH radical. The antiradical efficacy (AE) was calculated using the formula AE = 1/(EC_50_× TEC_50_) [114]. All experiments were performed in triplicate.

### 3.5. Synthesis

Triphenylantimony(V) catecholate complexes **1**–**8** were synthesized by an exchange reaction between the corresponding catechol-thioethers (1 mmol) and triphenylantimony(V) dibromide (1 mmol) in toluene solution (40 mL) in the presence of triethylamine (2 mmol). The reactants were dissolved in toluene and a solution (20 mL) of catechol-thioether was added to a solution (20 mL) of triphenylantimony(V) dibromide, followed by the addition of triethylamine. The reaction mixture was stirred for 1 h, and a precipitate of triethylammonium bromide was removed by filtration. Then the solvent was removed from a filtrate under reduced pressure and the residue was dissolved in hot n-hexane (~25 mL). The storage of solution at 0 °C overnight allowed us to obtain microcrystalline powders of catecholates **1**–**8**, which were collected by filtration and dried under reduced pressure. The yields were 71–83%.

#### 3.5.1. (3-(n-Butylthio)-4,6-di-tert-butyl-catecholato)triphenylantimony(V)

(3-BuS-4,6-Cat)SbPh_3_ (**1**). Yield 0.53 g (81%). IR (KBr, ν/cm^−1^): 3073, 3052, 2988, 2951, 2903, 2870, 1480, 1458, 1432, 1394, 1382, 1315, 1257, 1239, 1197. ^1^H-NMR (200 MHz, CDCl_3_, δ, ppm): 0.83 (t., ^3^J(H,H) = 7.3 Hz, 3 H, CH_3_), 1.20–1.38 (m., 2H, CH_2_), 1.45 (s., 9Н, tBu), 1.53 (s., 9Н, tBu), 1.56–1.68 (m., 2H, CH_2_), 2.88 (t., 3Н, ^3^J(H,H) = 7.6 Hz, 2H, SCH_2_), 6.76 (s., 1Н, arom. C_6_H_1_), 7.40–7.60 (m., 9H, Ph), 7.74–7.88 (m., 6H, Ph). ^13^C-NMR (50 MHz, CDCl_3_, δ, ppm): 13.80, 22.45, 29.62, 31.51, 31.59, 34.56, 34.71, 36.83, 112.99, 115.89, 129.18, 131.16, 132.23, 135.14, 137.81, 140.39, 142.97, 149.76. Elem. anal. calcd. for C_36_H_43_O_2_SSb (%): C, 65.36; H, 6.55; S, 4.85; Sb, 18.41; found (%): C, 65.41; H, 6.69; S, 4.82; Sb, 18.36.

#### 3.5.2. (3-(n-Hexylthio)-4,6-di-tert-butyl-catecholato)triphenylantimony(V)

(3-HexS-4,6-Cat)SbPh_3_ (**2**). Yield 0.55 g (80%). IR (KBr, ν/cm^−1^): 3071, 3060, 2987, 2951, 2907, 2873, 1478, 1458, 1432, 1394, 1382, 1315, 1260, 1241, 1201. ^1^H-NMR (200 MHz, CDCl_3_, δ, ppm): 0.86 (t., ^3^J(H,H) = 7.1 Hz, 3 H, CH_3_), 1.16–1.33 (m., 6H, CH_2_), 1.46 (s., 9Н, tBu), 1.54 (s., 9Н, tBu), 1.56–1.67 (m., 2H, CH_2_), 2.89 (t., 3Н, ^3^J(H,H) = 7.6 Hz, 2H, SCH_2_), 6.78 (s., 1Н, arom. C_6_H_1_), 7.45–7.55 (m., 9H, Ph), 7.80–7.88 (m., 6H, Ph). ^13^C-NMR (50 MHz, CDCl_3_, δ, ppm): 14.06, 22.58, 29.04, 29.47, 29.58, 31.47, 31.56, 34.69, 34.82, 36.81, 112.96, 115.87, 129.17, 131.16, 132.19, 135.13, 137.75, 140.32, 142.95, 149.71. Elem. anal. calcd. for C_38_H_47_O_2_SSb (%): C, 66.18; H, 6.87; S, 4.65; Sb, 17.66; found (%): C, 66.22; H, 7.05; S, 4.61; Sb, 17.60. 

#### 3.5.3. (3-(n-Octylthio)-4,6-di-tert-butyl-catecholato)triphenylantimony(V)

(3-OctS-4,6-Cat)SbPh_3_ (**3**). Yield 0.53 g (74%). IR (KBr, ν/cm^−1^): 3069, 3051, 2986, 2952, 2907, 2869, 1480, 1458, 1432, 1394, 1382, 1310, 1255, 1240, 1197. ^1^H-NMR (200 MHz, CDCl_3_, δ, ppm): 0.89 (t., ^3^J(H,H) = 6.4 Hz, 3 H, CH_3_), 1.14–1.35 (m., 10 H, CH_2_), 1.45 (s, 9 H, tBu), 1.53 (s, 9 H, tBu), 1.45–1.70 (m, 2 H, CH_2_), 2.87 (t, ^3^J(H,H) = 7.6 Hz, 2 H, SCH_2_), 6.76 (s, 1 H, arom. C_6_H_1_), 7.40–7.58 (m, 9H, Ph), 7.75–7.90 (m, 6H, Ph). ^13^C-NMR (50 MHz, CDCl_3_, ppm): 14.08, 22.63, 29.24, 29.32, 29.39, 29.53, 29.61, 31.50, 31.83, 34.71, 34.86, 36.82, 112.98, 115.93, 129.17, 131.15, 132.22, 135.15, 137.81, 140.39, 142.97, 149.74. Elem. anal. calcd. for C_40_H_51_O_2_SSb (%): C, 66.94; H, 7.16; S, 4.47; Sb, 16.97; found (%): C, 67.01; H, 7.41; S, 4.41; Sb, 16.84.

#### 3.5.4. (3-(Cyclopentylthio)-4,6-di-tert-butyl-catecholato)triphenylantimony(V)

(3-cycloPntS-4,6-Cat)SbPh_3_ (**4**). Yield 0.48 g (71%). IR (KBr, ν/cm^−1^): 2957, 2907, 2867, 1578, 1480, 1461, 1434, 1393, 1383, 1305, 1228, 1191. ^1^H-NMR (200 MHz, CDCl_3_, δ, ppm): 1.30–1.77 (m, 8 H, CH_2_), 1.45 (s, 9 H, tBu), 1.52 (s, 9 H, tBu), 3.96 (quint, ^3^J(H,H) = 6.4 Hz, 1 H, SCH), 6.76 (s, 1 H, arom. C_6_H_1_), 7.41–7.57 (m, 9H, Ph), 7.74–7.90 (m, 6H, Ph). ^13^C-NMR (50 MHz, CDCl_3_, δ, ppm): 24.85, 29.62, 31.65, 33.36, 34.68, 36.80, 46.02, 113.02, 116.27, 129.17, 131.13, 131.91, 135.02, 137.89, 140.30, 142.77, 149.33. Elem. anal. calcd. for C_37_H_43_O_2_SSb (%): C, 65.98; H, 6.43; S, 4.76; Sb, 18.08; found (%): C, 65.94; H, 6.49; S, 4.81; Sb, 18.13.

#### 3.5.5. (3-(Cyclohexylthio)-4,6-di-tert-butyl-catecholato)triphenylantimony(V)

(3-cycloHexS-4,6-Cat)SbPh_3_ (**5**). Yield 0.52 g (75%). IR (KBr, ν/cm^−1^): 2958, 2910, 2868, 1581, 1480, 1462, 1432, 1394, 1382, 1310, 1232, 1197. ^1^H-NMR (200 MHz, CDCl_3_, δ, ppm): 1.25–1.40 (m, 4 H, CH_2_), 1.46 (s, 9 H, tBu), 1.52 (s, 9 H, tBu), 1.45-1.70 (m, 4 H, CH_2_), 1.77–1.95 (m, 2 H, CH_2_), 3.55 (tt, ^3^J(H,H) = 11.4 Hz, ^3^J(H,H) = 3.5 Hz, 1 H, SCH), 6.77 (s, 1 H, arom. C_6_H_1_), 7.40-7.58 (m, 9H, Ph), 7.72–7.95 (m, 6H, Ph). ^13^C-NMR (50 MHz, CDCl_3_, δ, ppm): 25.94, 26.61, 29.62, 31.71, 33.73, 34.68, 36.81, 45.17, 113.15, 114.73, 129.18, 131.12, 131.90, 135.00, 137.87, 140.46, 142.78, 149.28. Elem. anal. calcd. for C_38_H_45_O_2_SSb (%): C, 66.38; H, 6.60; S, 4.66; Sb, 17.71; found (%): C, 66.43; H, 6.81; S, 4.65; Sb, 17.87.

#### 3.5.6. (3-(Benzylthio)-4,6-di-tert-butyl-catecholato)triphenylantimony(V)

(3-BzS-4,6-Cat)SbPh_3_ (**6**). Yield 0.55 g (79%). IR (KBr, ν/cm^−1^): 3072, 3045, 2954, 2907, 2873, 1578, 1495, 1480, 1456, 1431, 1394, 1357, 1312, 1258, 1238, 1169. ^1^H-NMR (200 MHz, CDCl_3_, δ, ppm): 1.49 (s, 9 H, tBu), 1.53 (s, 9 H, tBu), 4.09 (s, 2H, CH_2_), 6.80 (s, 1H, arom. C_6_H_1_), 7.15–7.35 (m, 5 H, Ph, Bz), 7.42–7.60 (m, 9H, Ph), 7.82–7.95 (m, 6H, Ph). ^13^C-NMR (50 MHz, CDCl_3_, δ, ppm): 29.62, 31.46, 34.77, 36.82, 39.15, 111.13, 115.51, 126.66, 128.24, 129.28, 131.25, 132.72, 135.14, 137.74, 138.55, 140.60, 143.14, 149.98. Elem. anal. calcd. for C_39_H_41_O_2_SSb (%): C, 67.34; H, 5.94; S, 4.61; Sb, 17.50; found (%): C, 67.31; H, 6.02; S, 4.60; Sb, 17.65.

#### 3.5.7. (3-(Phenylthio)-4,6-di-tert-butyl-catecholato)triphenylantimony(V)

(3-PhS-4,6-Cat)SbPh_3_ (**7**). Yield 0.55 g (80%). IR (KBr, ν/cm^−1^): 3068, 3045, 2957, 2910, 2871,1580, 1495, 1480, 1456, 1431, 1394, 1357, 1258, 1238, 1168. ^1^H-NMR (200 MHz, CDCl_3_, δ, ppm): 1.47 (s, 9 H, tBu), 1.51 (s, 9 H, tBu), 6.85 (s, 1H, arom. C_6_H_1_), 6.95–7.08 (m, 5 H, PhS), 7.30–7.47 (m, 9H, Ph), 7.48–7.58 (m, 6H, Ph). ^13^C-NMR (50 MHz, CDCl_3_, δ, ppm): 29.57, 31.44, 34.85, 36.69, 111.48, 113.22, 123.60, 125.75, 128.25, 129.03, 130.99, 133.67, 135.11, 137.33, 140.21, 141.48, 143.33, 149.90. Elem. anal. calcd. for C_38_H_39_O_2_SSb (%): C, 66.97; H, 5.77; S, 4.70; Sb, 17.87; found (%): C, 68.01; H, 5.96; S, 4.69; Sb, 17.81.

#### 3.5.8. (3-(Naphtyl-2-thio)-4,6-di-tert-butyl-catecholato)triphenylantimony(V)

(3-NapthS-4,6-Cat)SbPh_3_ (**8**). Yield 0.61 g (83%). IR (KBr, ν/cm^−1^): 3062, 3030, 2958, 2910, 2871, 1595, 1579, 1478, 1432, 1392, 1330, 1267, 1185. ^1^H-NMR (400 MHz, CDCl_3_, δ, ppm): 1.49 (s, 9 H, tBu), 1.54 (s, 9 H, tBu), 6.89 (s, 1 H, arom. C_6_H_1_), 7.16–7.22 (m, 7H, arom. C_10_H_7_), 7.31–7.39 (m, 5H, Ph), 7.40–7.49 (m, 9H, Ph), 7.67–7.72 (m, 1H, Ph). ^13^C-NMR (100 MHz, CDCl_3_, δ, ppm): 29.55, 31.45, 34.89, 36.72, 111.43, 113.18, 123.11, 124.33, 125.21, 125.88, 126.95, 127.52, 127.63, 128.22, 128.92, 129.03, 130.93, 131.01, 133.74, 133.91, 134.97, 135.24, 137.08, 137.86, 141.43, 143.21, 150.13. Elem. anal. calcd. for C_42_H_41_O_2_SSb (%): C, 68.95; H, 5.65; S, 4.38; Sb, 16.64; found (%): C, 68.92; H, 5.80; S, 4.37; Sb, 16.77.

## 4. Conclusions

Based on the exchange reaction of triphenylantimony(V) bromide with 3-RS-substituted 4,6-di-tert-butylcatechols, a series of new triphenylantimony(V) complexes **1**–**8** of the type (3-RS-4,6-DBCat)SbPh_3_ with catecholate ligands containing an additional redox center, a thioether group, were obtained. The crystal structures of catecholates with S-n-butyl-, S-n-hexyl-, S-n-octyl, and S-cyclohexyl groups (**1**–**3** and **5**, respectively) were characterized by single-crystal X-ray analysis. In crystals, molecules of **1** and **2** tend to form linear chains, while molecules of catecholate **3** form layers divided by n-octyl groups; complex **5** forms dimeric structures in crystalline state.

The investigation of the redox properties of complexes **1**–**8** by means of cyclic voltammetry showed that catecholate ligands, which are characterized by two oxidation stages, as well as a thioether linker, are involved in the electrochemical transformations. The shift in the potential of the first redox transition “catecholate/o-semiquinone” to the anodic region was associated with the electron-withdrawing effect of the RS group. At the same time, the nature of the substituent at the sulfur atom (alkyl, cycloalkyl, benzyl) had practically no significant effect on the oxidation potentials of the complexes. Only in the case of compounds **7** and **8** with aromatic groups ArS were shifts of the second and third oxidation potentials to the cathodic region, as well as partial stabilization of the doubly oxidized form of the complexes, observed.

The investigation of the antiradical properties of the complexes **1**–**8** in the reaction with DPPH radical showed that there is a decrease in the indicators of antiradical activity in comparison to the previously studied triphenylantimony (V) catecholates with donor alkoxy groups. This behavior could be explained by the presence of a thioether fragment that enhances the potential of the redox transition “catecholate/o-semiquinone”.

## Figures and Tables

**Table 1:** The selected bond lengths and valence angles in catecholates **1–3**, **5**.

Bond	1	2	3	5
	Distance, Å			
Sb(1)-O(1)	2.0272(12)	2.0487(15)	2.0519(14)	2.0124(10)
Sb(1)-O(2)	2.0374(12)	2.0196(15)	2.0175(13)	2.0536(10)
Sb(1)-C(x)	Sb(1)-C(19) 2.1005(18)	Sb(1)-C(15) 2.110(2)	Sb(1)-C(23) 2.140(2)	Sb(1)-C(21) 2.1438(15)
Sb(1)-C(y)	Sb(1)-C(31) 2.1391(19)	Sb(1)-C(21) 2.138(2)	Sb(1)-C(29) 2.1251(19)	Sb(1)-C(27) 2.1221(15)
Sb(1)-C(z)	Sb(1)-C(25) 2.1407(18)	Sb(1)-C(27) 2.141(2)	Sb(1)-C(35) 2.100(2)	Sb(1)-C(33) 2.1143(15)
O(1)-C(1)	1.362(2)	1.358(3)	1.355(2)	1.3740(17)
O(2)-C(2)	1.362(2)	1.354(2)	1.358(2)	1.3500(17)
C(1)-C(2)	1.406(2)	1.402(3)	1.403(3)	1.401(2)
C(1)-C(6)	1.395(2)	1.392(3)	1.400(3)	1.391(2)
C(2)-C(3)	1.394(2)	1.398(3)	1.397(3)	1.402(2)
C(3)-C(4)	1.417(2)	1.416(3)	1.418(3)	1.418(2)
C(4)-C(5)	1.401(2)	1.403(3)	1.399(3)	1.400(2)
C(5)-C(6)	1.399(2)	1.404(3)	1.400(3)	1.404(2)
S(1)-C(3)	1.7903(17)	1.776(2)	1.785(2)	1.7801(15)
S(1)-C(7)	1.8227(19)	1.826(12)	1.829(2)	1.8343(16)
	Angle, °			
	O(1)-Sb(1)-O(2) 79.01(5)	O(1)-Sb(1)-O(2) 78.91(6)	O(1)-Sb(1)-O(2) 78.90(5)	O(1)-Sb(1)-O(2) 78.87(4)
	O(1)-Sb(1)-C(31) 151.25(6)	O(2)-Sb(1)-C(21) 145.63(7)	O(2)-Sb(1)-C(29) 142.51(7)	O(2)-Sb(1)-C(27) 128.62(5)
	O(2)-Sb(1)-C(25) 150.38(6)	O(1)-Sb(1)-C(27) 156.27(8)	O(1)-Sb(1)-C(23) 158.06(7)	O(1)-Sb(1)-C(21) 162.40(5)

**Table 2:** The cyclic voltammetry (CV) data for **1**–**8** (*vs.* Ag/AgCl/KCl(sat.)).

№	Complex	Е^ox1^_1/2_, V	I_с_/I_а_	Е^ox2^_p_, V	Е^ox3^_1/2_, V
**1**	(3-BuS-4,6-Cat)SbPh_3_	0.94	0.74	1.40	1.69
**2**	(3-HexS-4,6-Cat)SbPh_3_	0.96	0.82	1.43	1.71
**3**	(3-OctS-4,6-Cat)SbPh_3_	0.94	0.75	1.44	1.70
**4**	(3-cycloPntS-4,6-Cat)SbPh_3_	0.94	0.85	1.45	1.69
**5**	(3-cycloHexS-4,6-Cat)SbPh_3_	0.94	0.73	1.44	1.69
**6**	(3-BzS-4,6-Cat)SbPh_3_	0.95	0.80	1.43	1.72
**7**	(3-PhS-4,6-Cat)SbPh_3_	0.96	0.83	1.38	1.70
**8**	(3-NapthS-4,6-Cat)SbPh_3_	0.96	0.92	1.32	1.58

Conditions: under argon, CH_2_Cl_2_ solution containing 0.15 M [N(nBu)_4_]ClO_4_ supporting electrolyte, glassy carbon working electrode; scan rate 200 mV∙s^−1^; *С* = 3∙10^−3^ M. Potentials are given vs. Ag/AgCl/KCl(sat.). Notes: Е^ox1^_1/2_ and Е^ox3^_1/2_-half-wave potentials of the first and third anodic processes; Е^ox2^_p_-peak potential of the second anodic process; I_с_/I_а_-current ratios.

**Table 3:** Indicators of antiradical activity of **1**–**8** in the test with the DPPH radical (CH_2_Cl_2_, 298 K).

№	Complex	ЕС_50_, μmol/L	TEC_50_, Min	AE·10^3^
**1**	(3-BuS-4,6-Cat)SbPh_3_	29.5 ± 1.2	170	0.20
**2**	(3-HexS-4,6-Cat)SbPh_3_	32.4 ± 0.9	180	0.17
**3**	(3-C_8_H_17_S-4,6-Cat)SbPh_3_	32.0 ± 1.0	180	0.17
**4**	(3-C_5_H_9_S-4,6-Cat)SbPh_3_	29.2 ± 1.1	130	0.26
**5**	(3-C_6_H_11_S-4,6-Cat)SbPh_3_	26.3 ± 1.0	130	0.29
**6**	(3-BzS-4,6-Cat)SbPh_3_	31.7 ± 1.4	170	0.19
**7**	(3-PhS-4,6-Cat)SbPh_3_	31.0 ± 0.8	180	0.18
**8**	(3-NapthS-4,6-Cat)SbPh_3_	30.5 ± 0.9	170	0.19
	(3,6-Cat)SbPh_3_ [75]	17.3 ± 0.5	60	0.96
	(4-MeO-3,6-Cat)SbPh_3_ [75]	6.8 ± 0.8	22	6.68

## Data Availability

The data presented in this study are available in this article.

## Supplementary Materials

The following are available online. Table S1: Crystal data and structure refinement for 1, 2∙0.5 Toluene, 3, 5; Figures S1–S4: Fragments of the crystal packing of 1, 2, 3, 5; Figures S5–S12: The CVs of complexes. CCDC 2071115 (1), 2071116 (2∙0.5Toluene), 2071117 (3), 2071118 (5) contain the supplementary crystallographic data for this paper. These data can be obtained free of charge from The Cambridge Crystallographic Data Centre via www.ccdc.cam.ac.uk/data_request/cif (accessed on 26 February 2021).

## Sample Availability

Samples of the compounds **1**–**8** are available from the authors.
