# Supplementary material for: Triphenylantimony(V) Catecholates of the Type (3-RS-4,6-DBCat)SbPh3-Catechol Thioether Derivatives: Structure, Electrochemical Properties, and Antiradical Activity
Source: Molecules. 2021 Apr 9;26(8):2171. doi: 10.3390/molecules26082171 (PMC8069174; doi:10.3390/molecules26082171)
Supplement: Supplementary file 1 [file molecules-26-02171-s001.pdf]

Supplementary materials

Triphenylantimony(V) Catecholates of the type (3-SR-4,6-DBCat)$SbPh_3$ - Catechol Thioether Derivatives: Structure, Electrochemical Properties and Antiradical Activity

Ivan V. Smolyaninov[1], Georgy K. Fukin[2], Nadezhda T. Berberova[1] and Andrey I. Poddel'sky[2,*]

[1] Astrakhan State Technical University, Department of Chemistry, 16 Tatisheva str., Astrakhan 414056, Russian Federation; ivsmolyaninov@gmail.com (I.S.); nberberova@gmail.com (N.B.)

[2] G.A. Razuvaev Institute of Organometallic Chemistry, Russian Academy of Sciences, 49 Tropinina str., 603137 Nizhny Novgorod, Russian Federation; aip@iomc.ras.ru (A.P.); gera@iomc.ras.ru (G.F.)

[*] Correspondence: aip@iomc.ras.ru; Tel.(Fax): +7-831- 462-7497

Content

Table S1. Crystal data and structure refinement for **1**, **2**·0.5 Toluene, **3**, **5** 2

Figure S1. A fragment of the crystal packing of (6-BuS-3,5-DBCat)$SbPh_3$ (**1**) 3

Figure S2. A fragment of the crystal packing of (6-HexS-3,5-DBCat)$SbPh_3$ (**2**) 3

Figure S3. A fragment of the crystal packing of (6-OctS-3,5-DBCat)$SbPh_3$ (**3**) 4

Figure S4. A fragment of the crystal packing of (6-cycloHexS-3,5-DBCat)$SbPh_3$ (**5**) 4

Figure S5. The CVs of the oxidation of **1** in potential ranges from -0.60 to 1.20 V and from -0.60 to 1.90 V 5

Figure S6. The CVs of the oxidation of **2** in potential ranges from -0.68 to 1.25 V and from -0.60 to 1.90 V 5

Figure S7. The CVs of the oxidation of **3** in potential ranges from -0.68 to 1.20 V and from -0.68 to 1.90 V 6

Figure S8. The CVs of the oxidation of **4** in potential ranges from -0.67 to 1.20 V and from -0.67 to 1.90 V 6

Figure S9. The CVs of the oxidation of **5** in potential ranges from -0.67 to 1.20 V and from -0.70 to 1.90 V 7

Figure S10. The CVs of the oxidation of **5** in potential ranges from -0.70 to 1.20 V (5 scans) 7

Figure S11. The CV curves of the oxidation of **6** in potential ranges from -0.68 to 1.20 V and from -0.68 to 1.85 V 8

Figure S12. The CV curves of the oxidation of **8** in potential ranges from -0.70 to 1.15 V and from -0.70 to 1.80 V 8

Table S1. Crystallographic and refinement data for **1**, **2**·0.5 Toluene, **3**, **5**.

Complex	**1**	**2**·0.5 Toluene	**3**	**5**
Empirical formula	$C_{36}H_{43}O_2SSb$	$C_{41.5}H_{51}O_2SSb$	$C_{40}H_{51}O_2SSb$	$C_{38}H_{45}O_2SSb$
Formula weight	661.51	735.63	717.61	687.55
T/K	100(2)	100(2)	100(2)	100(2)
Crystal system	Monoclinic	Monoclinic	Triclinic	Triclinic
Space group	P2(1)/n	P2(1)/n	P-1	P-1
a/Å	17.1736(12)	13.1137(3)	10.7382(4)	11.48922(17)
b/Å	9.7483(9)	17.6440(4)	12.7668(5)	13.26631(15)
c/Å	19.1115(13)	16.0472(4)	14.4074(6)	13.7347(3)
α/°	90	90	79.9850(10)	109.6728(15)
β/°	90.826(2)	101.63	78.0380(10)	107.2475(15)
γ/°	90	90	75.0250(10)	106.4133(12)
$V/Å^{-3}$	3199.2(4)	3636.73(15)	1851.44(13)	1703.94(5)
Z	4	4	2	2
$\rho/g\cdot cm^{-3}$	1.373	1.344	1.287	1.340
μ, mm^{-1}	0.957	0.850	0.833	0.902
Q range/°	1.606 - 28.996	1.961 - 25.999	1.457 - 28.700	3.032 - 29.999
Reflections collected	24290	31277	19932	54603
Unique reflections collected	8455	7140	9511	9837
R_{int}	0.0213	0.0247	0.0236	0.0390
Parameters	1368	1532	748	385
R_1 / wR_2 [I>2σ(I)]	0.0295 / 0.0726	0.0293 / 0.0759	0.0325 / 0.0740	0.0254 / 0.0538
R_1 / wR_2 (all data)	0.0348 / 0.0754	0.0344 / 0.0791	0.0370 / 0.0768	0.0332 / 0.0555
GOOF(F^2)	1.043	1.066	1.044	1.055
Largest diff. peak and hole/ $e\cdot Å^{-3}$, ρ_{max}/ρ_{min}	1.531 / -0.399	1.179 / -0.806	1.247 / -0.406	0.662 / -0.392

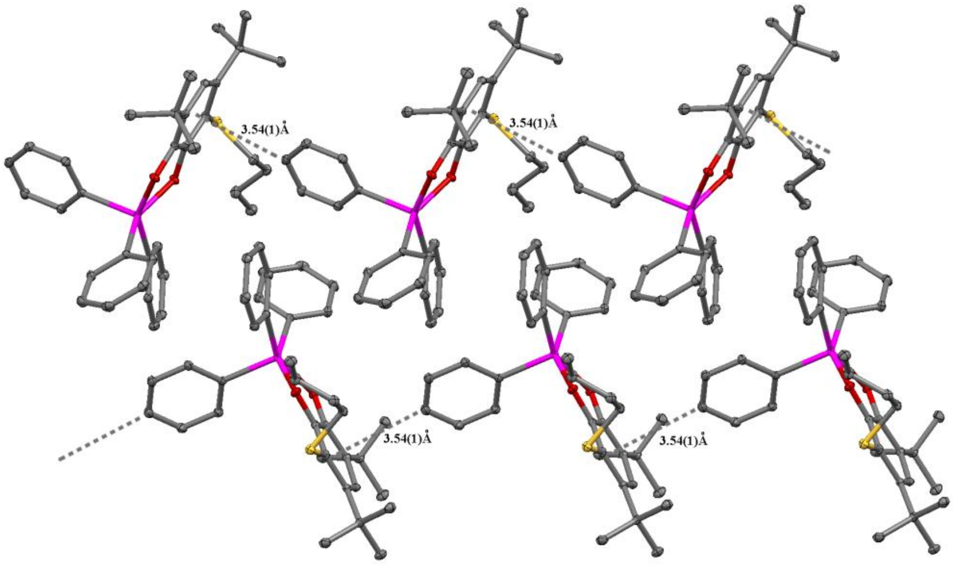

Figure S1. A fragment of the crystal packing of (6-BuS-3,5-DBCat)$SbPh_3$ (**1**) with an indication of the "C_{Ph}...π-system$_{Cat}$" interactions between molecules (hydrogen atoms are not shown).

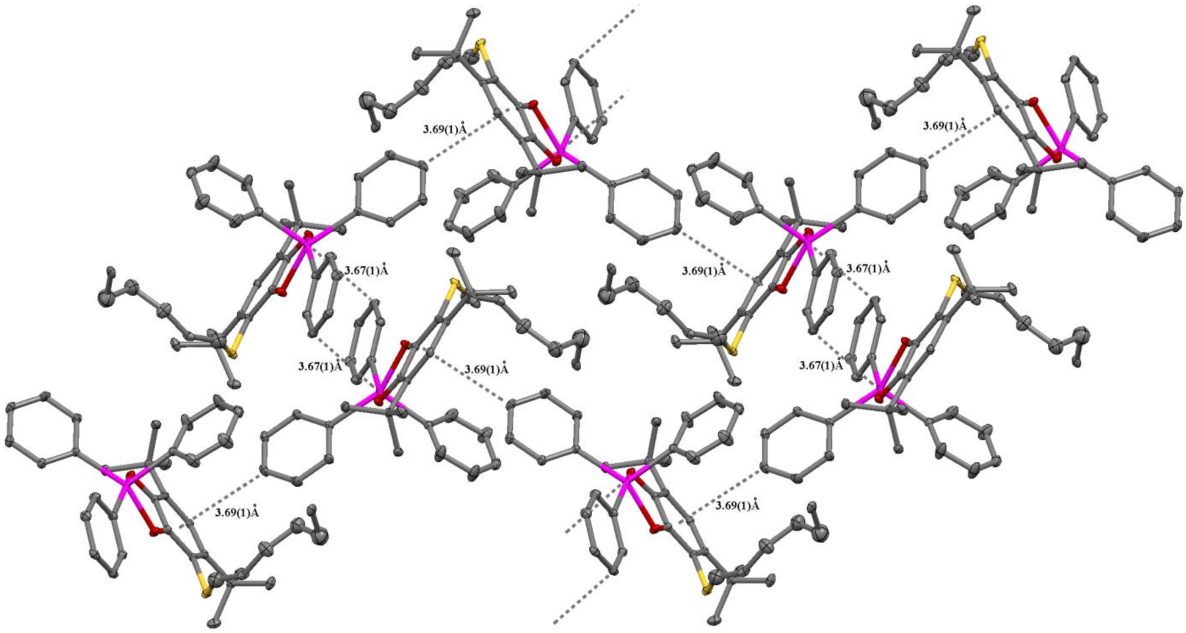

Figure S2. A fragment of the crystal packing of (6-HexS-3,5-DBCat)$SbPh_3$ (**2**) with an indication of the "C_{Ph}...π-system$_{Cat}$" and "Sb...C_{Ph}" interactions between molecules (hydrogen atoms are not shown).

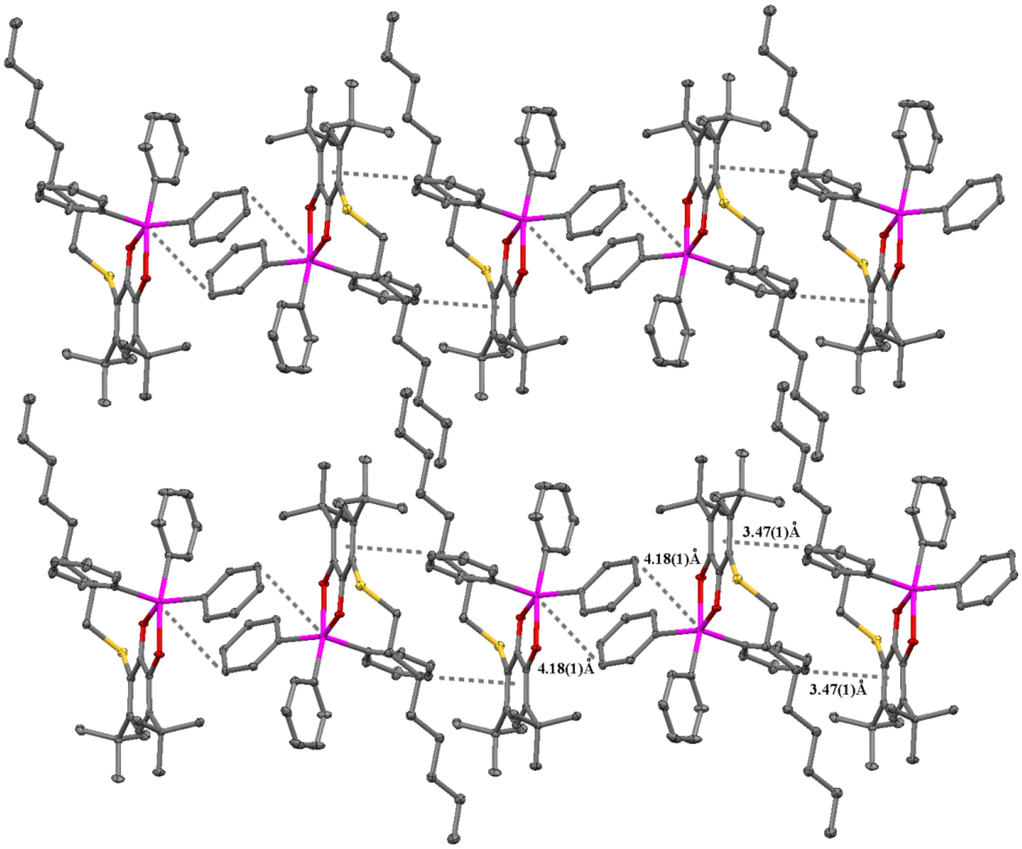

Figure S3. A fragment of the crystal packing of (6-OctS-3,5-DBCat)$SbPh_3$ (**3**) with an indication of the "C_{Ph}...π-system$_{Cat}$" and "Sb...C_{Ph}" interactions between molecules (hydrogen atoms are not shown).

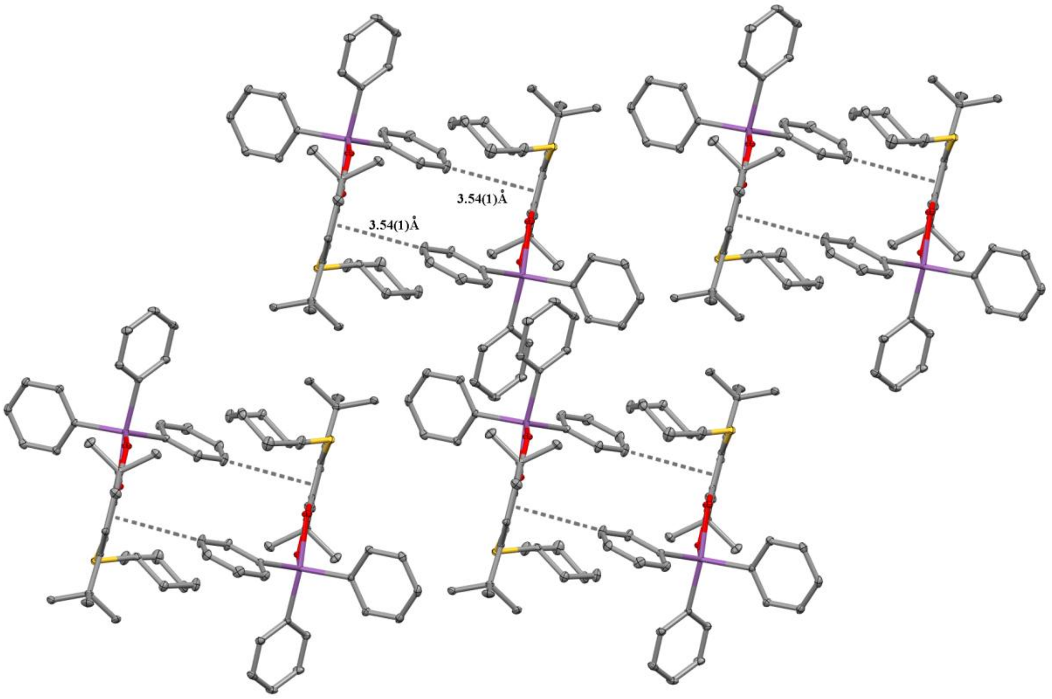

Figure S4. A fragment of the crystal packing of (6-cycloHexS-3,5-DBCat)$SbPh_3$ (**5**) with an indication of the "C_{Ph}...π-system$_{Cat}$" interactions between molecules (hydrogen atoms are not shown).

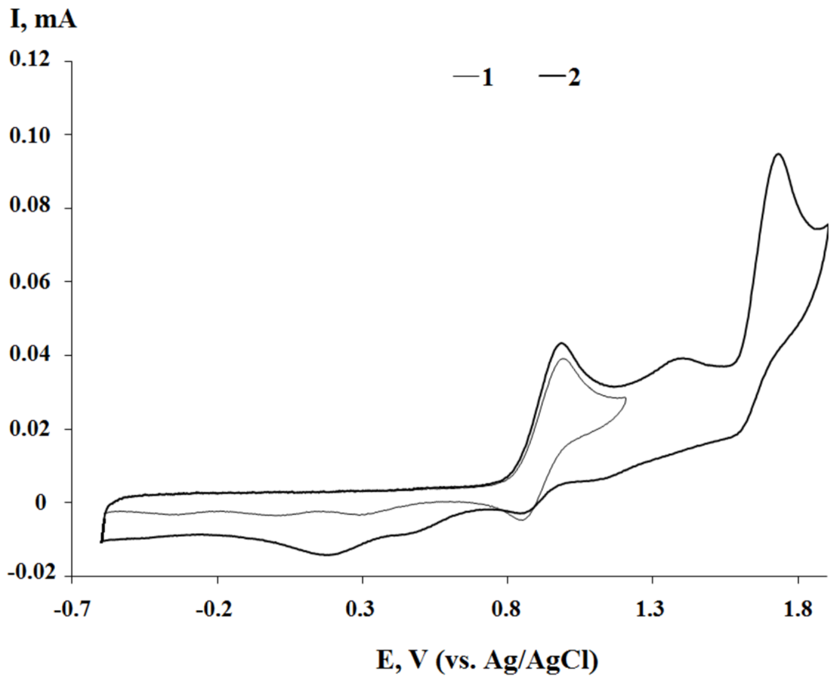

Figure S5. The CV curves of the oxidation of **1** in potential ranges from -0.60 to 1.20 V (curve 1); from -0.60 to 1.90 V (curve 2) (CH_2Cl_2, GC anode, Ag/AgCl/KCl(sat.), 0.15M $[nBu_4N]ClO_4$, C = $3{\cdot}10^{-3}$ M, argon).

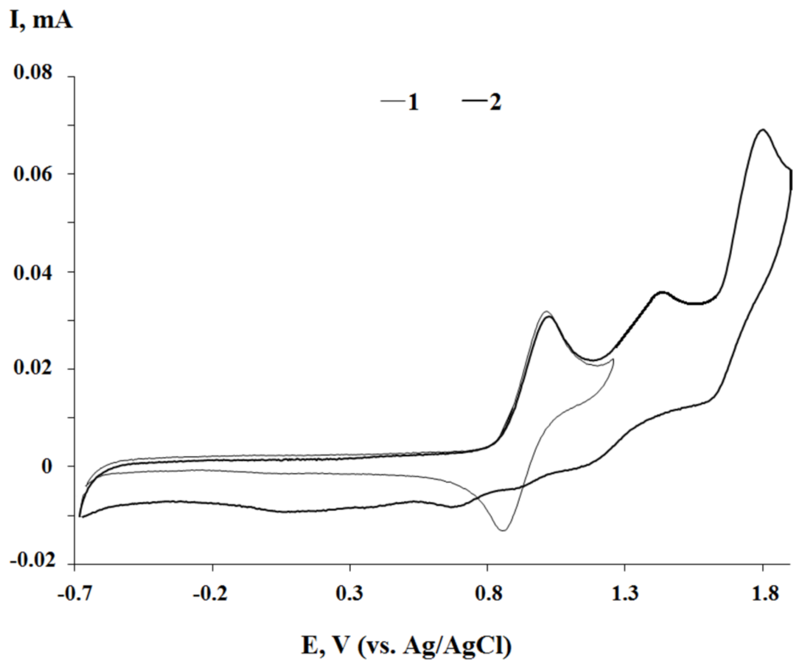

Figure S6. The CV curves of the oxidation of **2** in potential ranges from -0.68 to 1.25 V (curve 1); from -0.60 to 1.90 V (curve 2) (CH_2Cl_2, GC anode, Ag/AgCl/KCl(sat.), 0.15M $[nBu_4N]ClO_4$, C = $2{\cdot}10^{-3}$ M, argon).

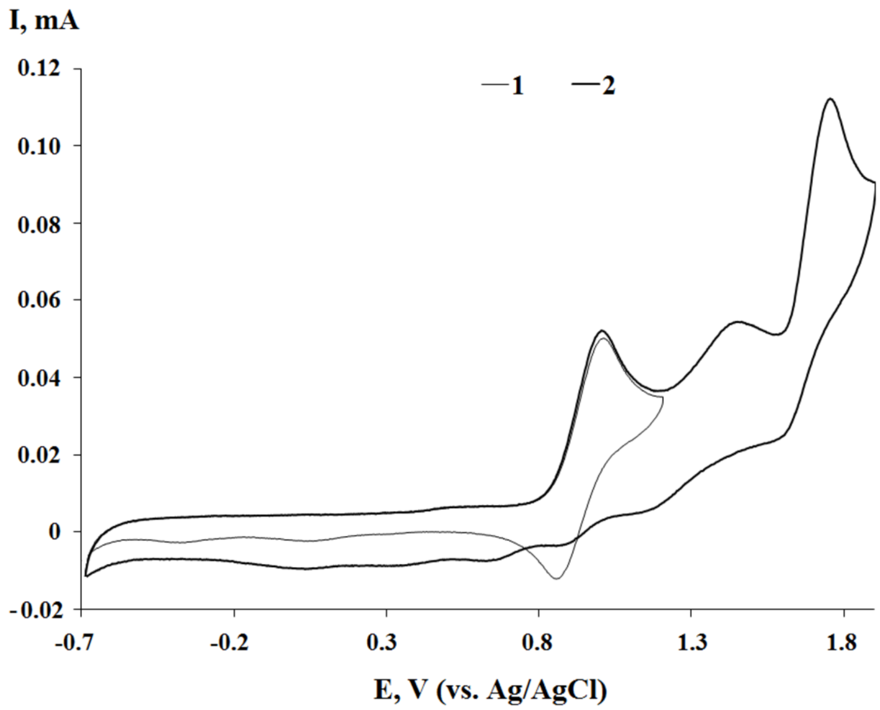

Figure S7. The CV curves of the oxidation of **3** in potential ranges from -0.68 to 1.20 V (curve 1); from -0.68 to 1.90 V (curve 2) (CH_2Cl_2, GC anode, Ag/AgCl/KCl(sat.), 0.15M $[nBu_4N]ClO_4$, C = $3{\cdot}10^{-3}$ M, argon).

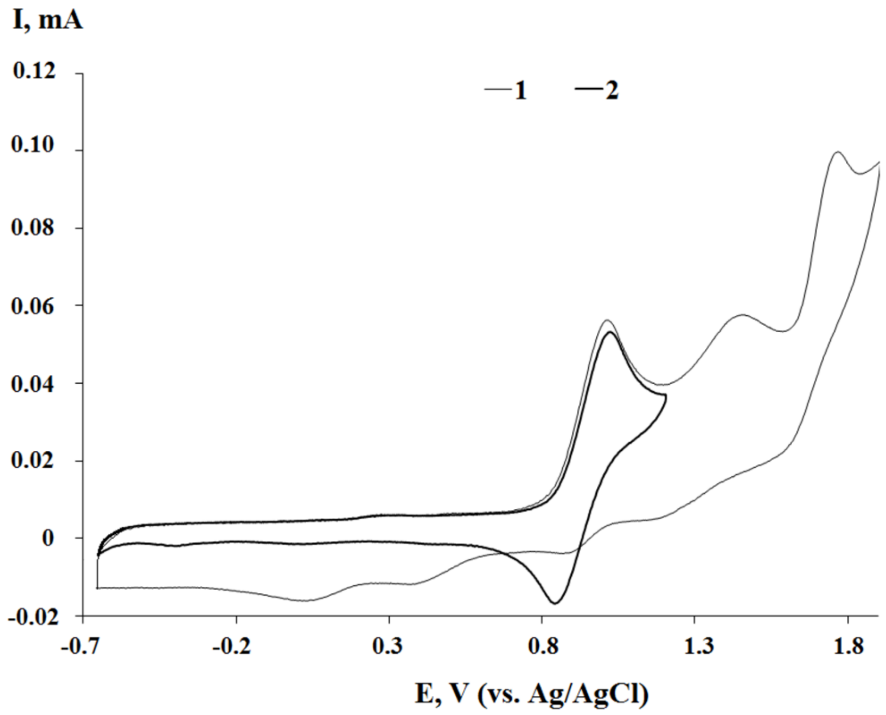

Figure S8. The CV curves of the oxidation of **4** in potential ranges from -0.67 to 1.20 V (curve 1); from -0.67 to 1.90 V (curve 2) (CH_2Cl_2, GC anode, Ag/AgCl/KCl(sat.), 0.15M $[nBu_4N]ClO_4$, C = $3{\cdot}10^{-3}$ M, argon).

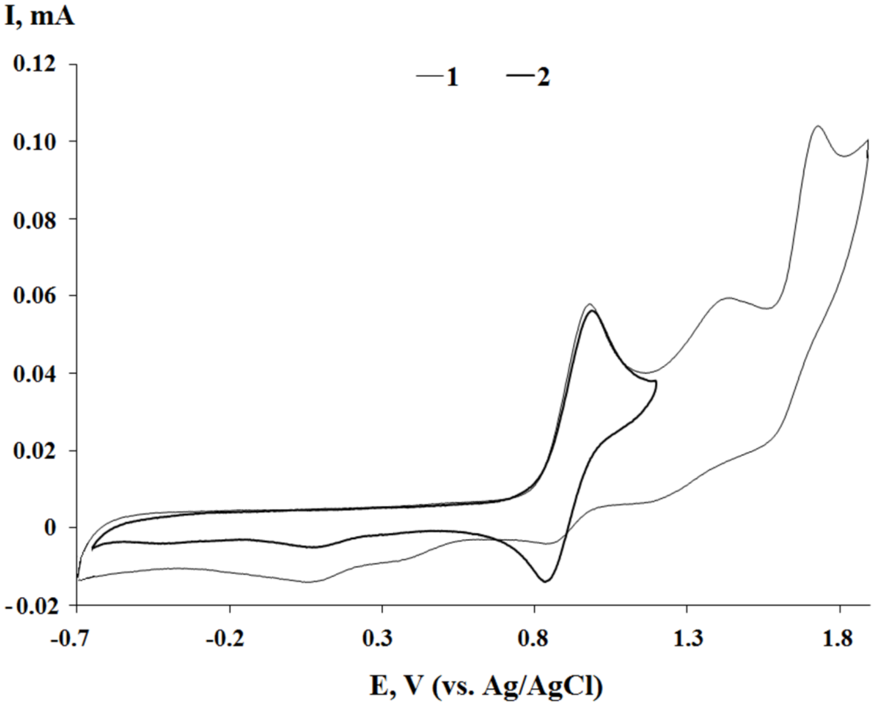

Figure S9. The CV curves of the oxidation of **5** in potential ranges from -0.67 to 1.20 V (curve 1); from -0.70 to 1.90 V (curve 2) (CH_2Cl_2, GC anode, Ag/AgCl/KCl(sat.), 0.15M $[nBu_4N]ClO_4$, C = $3{\cdot}10^{-3}$ M, argon).

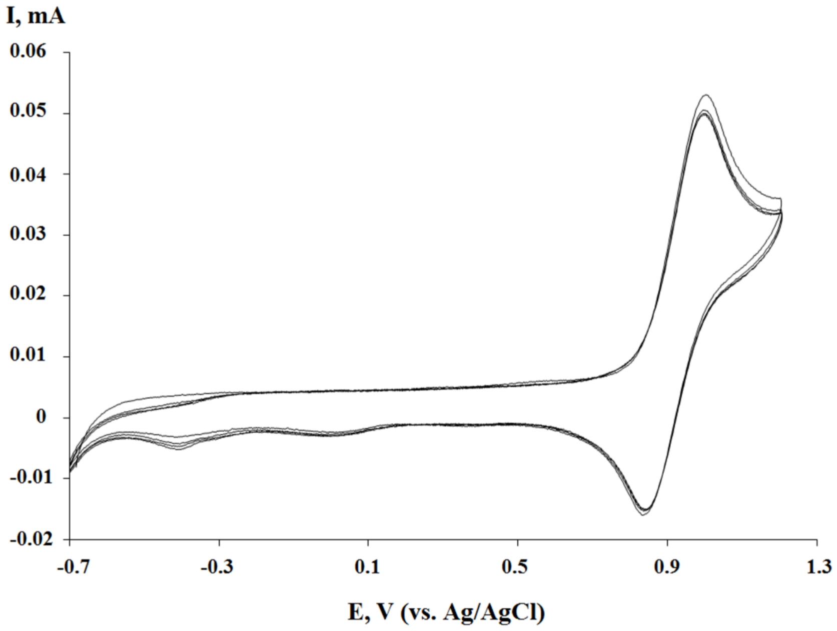

Figure S10. The CV curves of the oxidation of **5** in potential ranges from -0.70 to 1.20 V (5 scans) (CH_2Cl_2, GC anode, Ag/AgCl/KCl(sat.), 0.15M $[nBu_4N]ClO_4$, C = $3{\cdot}10^{-3}$ M, argon).

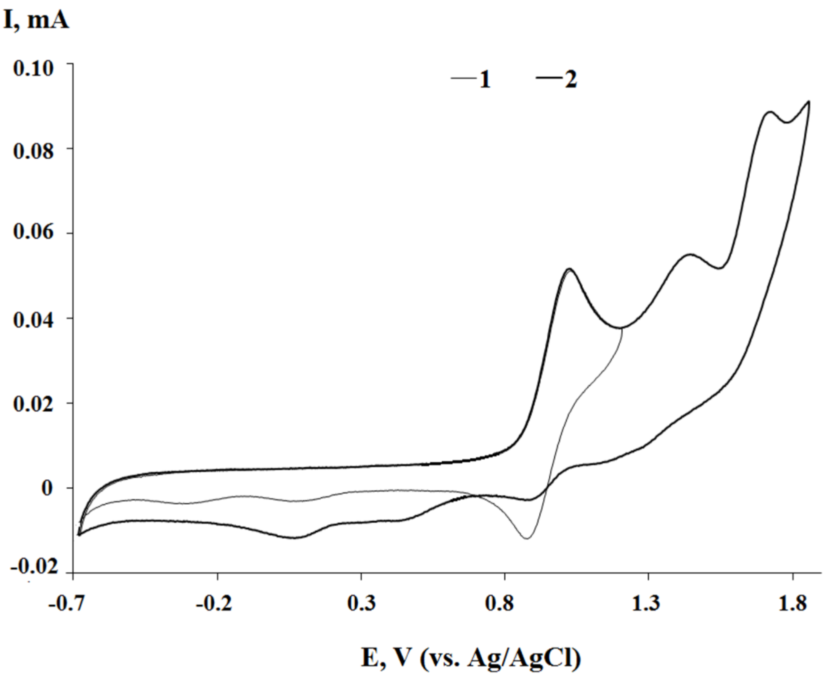

Figure S11. The CV curves of the oxidation of **6** in potential ranges from -0.68 to 1.20 V (curve 1); from -0.68 to 1.85 V (curve 2) (CH_2Cl_2, GC anode, Ag/AgCl/KCl(sat.), 0.15M $[nBu_4N]ClO_4$, C = $3{\cdot}10^{-3}$ M, argon).

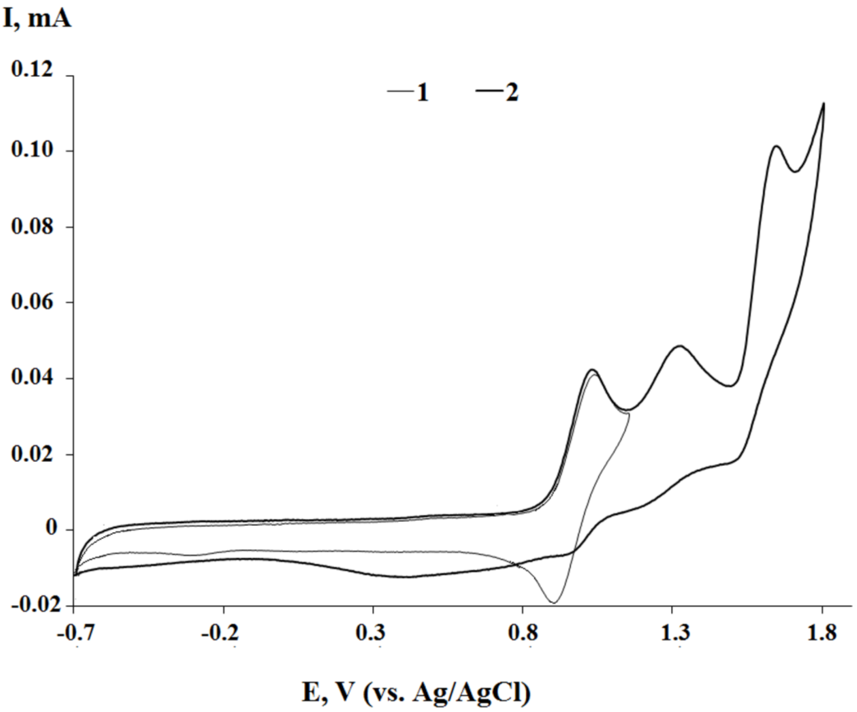

Figure S12. The CV curves of the oxidation of **8** in potential ranges from -0.70 to 1.15 V (curve 1); from -0.70 to 1.80 V (curve 2) (CH_2Cl_2, GC anode, Ag/AgCl/KCl(sat.), 0.15M $[nBu_4N]ClO_4$, C = $3{\cdot}10^{-3}$ M, argon).
